# Editorial: Quinoline and *N*-heterocycle analogues as lead structures for the development of antiprotozoal agents

**DOI:** 10.3389/fchem.2026.2005043

**Published:** 2026-10-02

**Authors:** Angel H. Romero, Gustavo Benaim, Jaime Charris, Hugo Cerecetto

**Affiliations:** 1 Grupo de Química Orgánica Medicinal, Facultad de Ciencias, Universidad de La República, Montevideo, Uruguay; 2 Instituto de Investigación Una Salud, Universidad de La República, Montevideo, Uruguay; 3 Unidad de Señalización Celular y Bioquímica de Parásitos, Instituto de Estudios Avanzados (IDEA), Caracas, Venezuela; 4 Instituto de Biología Experimental, Facultad de Ciencias, Universidad Central de Venezuela, Caracas, Venezuela; 5 Laboratorio de Síntesis Orgánica, Facultad de Farmacia, Universidad Central de Venezuela, Caracas, Venezuela; 6 Área de Radiofarmacia, Centro de Investigaciones Nucleares, Facultad de Ciencias, Universidad de La República, Montevideo, Uruguay

**Keywords:** antiprotozoal agents, hexahydroquinoline, leishmaniasis, malaria, quinoline, trypanosomiasis

Tropical protozoan diseases constitutes a group of endemic conditions primarily present in low-income countries of tropical and subtropical regions of Africa, Asia and America (Lupi et al., 2009; Bright-Nwoke and Nneka-Ukaga, 2014). They are caused by a variety of single-celled protozoan, which are transmitted to humans by insect vectors with complex life cycles (Lupi et al., 2009; Bright-Nwoke and Nneka-Ukaga, 2014). Major examples include malaria, Chagas disease, leishmaniasis and human African trypanosomiasis (HAT), which are caused by *Plasmodium* spp., *T. cruzi*, *Leishmania* spp. and *T. brucei* parasites, respectively (Lupi et al., 2009; Bright-Nwoke and Nneka-Ukaga, 2014; von Stebut, 2022). In general, tropical diseases caused by protozoa affect about 1 billion people worldwide, registering in high morbidity and mortality rates (World Health Organization, 2024). Due to the lack of vaccination regimens, drug-therapies remain the only solutions. Treatment remains a challenge, as existing therapies are outdated and present significant drawbacks including a high degree of toxicity, high cost, low therapeutic efficacy, poor bioavailability, problematic administration regimens and, parasite resistance (Muschietti et al., 2013). Therefore, there is an urgent need to identify lead structures for the rational design of safer and more potent antiprotozoal agents.

Historically, quinoline has been used as a scaffold for the development of antimalarial agents with a well-defined mechanism based on the inhibition of *β*-hematine polymerization (Foley and Tilley, 1998). In recent decades, quinoline antimalarial drugs have been employed in repurposing program for treatment of leishmaniasis (Avanzo et al., 2025; Silva et al., 2024). In particular, the 4-and 8-aminoquinolines represent a promising platform for the design of new leishmanicidal agents targeting toward alteration of mitochondrial functions and immunostimulation of host-cell (Romero and Delgado; Del Carpio et al., 2025). Against Chagas Disease, quinoline has emerged as an attractive platform to disrupt heme metabolism in infected models, thereby impairing the survival of the intracellular stage (Gorka et al., 2013). Meanwhile, quinoline amides have been identified as a scaffold for further optimization against HAT (Koester et al., 2022). Other *N*-heterocycle analogues-such as quinazoline, pteridine, isoquinoline, quinoxaline and acridine-form part of many of the most promising antiprotozoal agents (Maheshwari and Bandyopadhyay, 2021). Considering the importance of quinolines and related *N*-heterocycles, the current Research Topic presents a collection of seven articles, discussing: i) the role of the substitution site and the use of natural products in the leishmanicidal activity of quinolines (3 articles); ii) the potential of 4-substituted quinolines as antimalarials agents (2 articles), iii) the chemistry and therapeutic potential of hexahydroquinolines as antiprotozoan agent (1 article) and iv) a perspective toward translational research in diseases caused by trypanosomatids (1 article).

Regarding the leishmaniasis Research Topic, the first article, a review article (Romero and Delgado) analyzed the potential of 4-aminoquinolines as a privileged scaffold for the design leishmanicidal agents. The authors discussed how the incorporation of basic groups (preferably tertiary amines) and lipophilic moieties modulates both the selectivity and potency of quinoline compounds. The review highlighted that a substituted-aniline moiety at the 4-position significantly enhances the leishmanicidal potency compared to an alkylamine chain. Mechanistic aspects were also addressed, indicating that 4-aminoquinolines promote: i) the depolarization of parasite´s mitochondrial potential, thereby inducing apoptosis and morphologic changes and, ii) the immunostimulation of infected macrophages through a substantial increase in proinflammatory cytokine and nitric oxide production. Promising 4-aminoquinoline-based leishmanicidal candidates (**1–7)** for preclinical studies are illustrated in Figure 1A.

**FIGURE 1:**
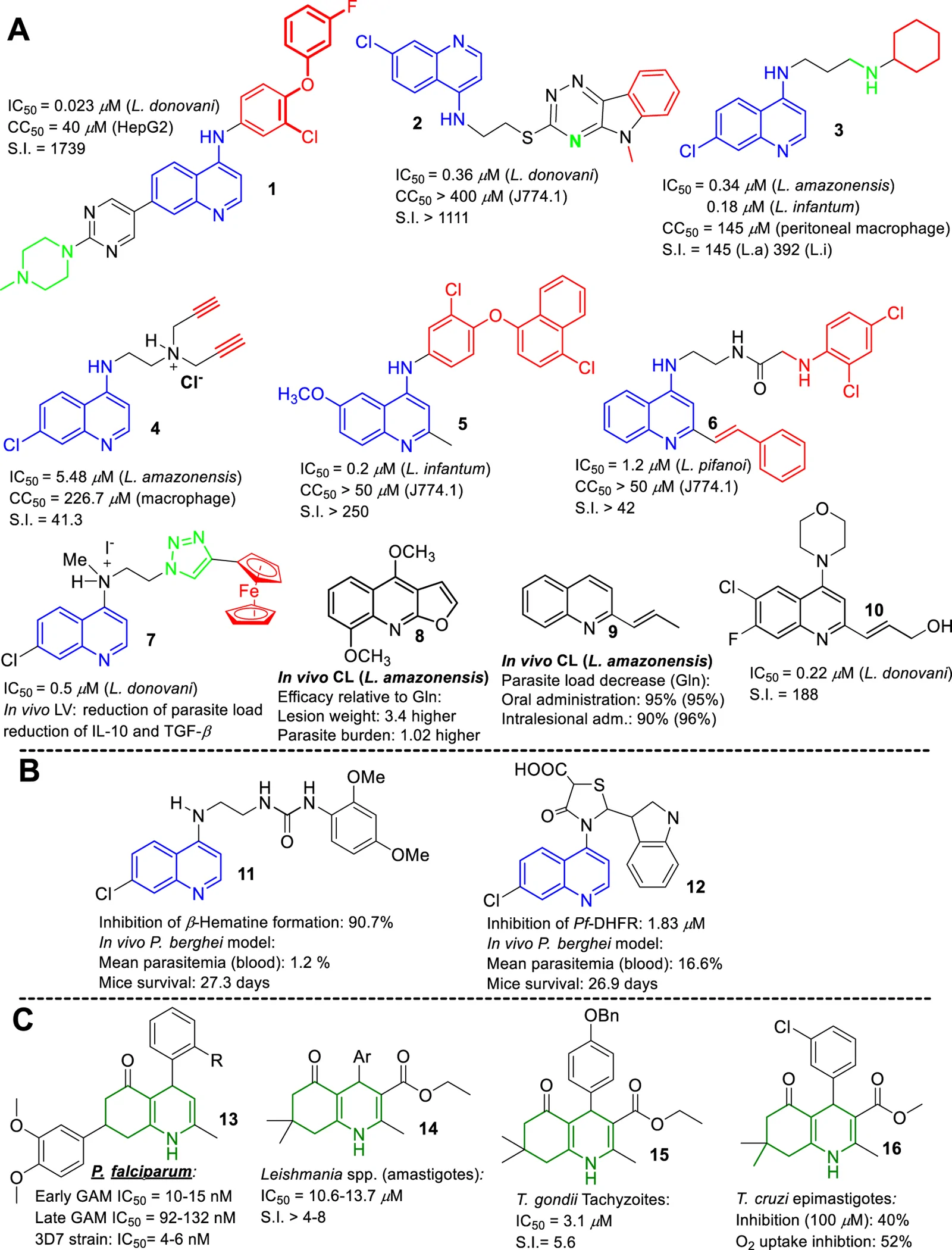
Promising antiprotozoal quinoline candidates (**1–16**) for preclinical studies. **(A)** shows chemical structures of quinoline derivatives numbered 1 to 10, each color-coded to highlight functional groups, with associated antileishmanial activity data and selectivity indices. **(B)** presents compounds 11 and 12 with structures and detailed antimalarial efficacy information, including inhibition, parasitemia, and mouse survival data. **(C)** contains four hexahydroquinolines, 13 to 16, with green- highlighted features, indicating their inhibitory concentrations and selectivity for malaria, *leishmania, toxoplasma gondii*, and *trypanosoma cruzi*, accompanied by IC_50_ values and inhibition percentages.

The second report, a minireview (Yaluff et al., 2025) compiled the therapeutic potential of quinoline-based natural products against various *in vivo* leishmanicial models. These examples show that most of isolated quinoline secondary metabolites exhibiting antileishmania bioactivity are 2-alkylquinolines, highlighting that the incorporation of either an alkyl chain or an aryl chain is key to generate active and potent leishmanicidal agents (**8** and **9**, Figure 1A). This active 2-alkylquinoline **9** served as structural platform for the design of synthetic 2-substituted quinolines, such as compound **10** (Garcia and Elso).

The third report, a minireview (Garcia and Elso) discusses the role of 2-, 3-, 6- and 8-substitution in the design of leishmanicidal quinolines. The report shows that the inclusion of lipophilic chains like alkenyl or aryl at the 2-, 3- and 6-position significantly favors both leishmanicidal activity and selectivity (**10**, Figure 1A), whereas the incorporation of *O*- or *N*-substituent is preferred at the 8-position. Meanwhile, *N*-substitution at the 2 and 3-positions yields compounds that exhibit only a modest leishmanicidal response.

Concerning the antimalarial Research Topic, Ramirez and co-workers (Ramirez et al.) reported the potential of a series of novel 4-substituted 7-chloroquinolines (22 derivatives) bearing urea, carbamate or thiourea functional groups. Initially, eight compounds demonstrated significant inhibition of *β*-hematin formation. Notably, compound **11** (Figure 1B) exhibited an inhibition of approximately 90.7%, which is highly comparable to that of the reference chloroquine (92.6%). Further evaluation using an *in vivo Plasmodium berghei* model showed that compound **11** reduced parasitemia to 1.2%, with an extended survival time (27.3 days), which was comparable to therapeutic response of the chloroquine reference. Additionally, the compound displayed low hematotoxicity against human erythrocytes.

The second report regarding antimalarials describes the synthesis, molecular docking and biological evaluation of a series of novel 4-aminoquinoline-thiazolidinone hybrids (12 compounds) against malarial models (Fatima et al.). Five of these compounds demonstrated significant inhibition of the *Pf-*DHFR enzyme (IC_50_ < 2 *µ*M), exhibiting potencies comparable to that of the reference drug. Four compounds, most notably compound **12** (Figure 1B), showed excellent activity against an *in vivo P. berghei* model, achieving a reduction in parasitemia to 16.6%, with significant animal survival (26.9 days). Molecular studies revealed a favourable binding accommodation within the Pf-DHFR active site, while *in silico* profiling confirmed the security of the compounds in terms of keys medicinal parameters.

On the other hand, Odeselu and co-workers reviewed the therapeutic potential of the hexahydroquinoline core as antiprotozoal agents (Oduselu et al.). Synthetic strategies for preparation of both 1,4,5,6,7,8-hexahydroquinoline and 1,2,3,4,4a,5-hexahydroquinoline were discussed in detail. In particular, the authors highlighted the biological relevance of 1,4,5,6,7,8-hexahydro-6-oxo-3-carboxylquinolines **13–16** (Figure 1C) against *in vitro* model of *Plasmodium falciparum* (malaria), *leishmania* spp (leishmaniasis), *Trypanosoma cruzi* (Chagas disease) and *Toxoplasma gondii* (Toxoplasmosis). These scaffolds were identified as promising structural platforms for future drug design.

Finally, Ponte-Sucre discussed the ongoing challenge in identifying effective strategies to achieve universal access to high-quality diagnostics and therapeutic options for diseases caused by protozoa. Author reflects that while considerable progress has been made in the diagnosis, prevention, and treatment of trypanosomatidae-transmitted diseases, major efforts must remain focused on the development of novel drugs. Article highlights that more robust strategies are required to better integrate computational data with experimental findings, thereby accelerating the discovery of a new antiprotozoal drug portfolio.

In summary, the review, minireviews and original research articles compiled in this Research Topic collectively highlight the potential of the quinoline core as a privileged scaffold for the design of potent and selective antiprotozoal agents. Comprehensive insights into structure-activity, the specific roles of the quinoline-substitution patterns, mechanisms of action, immunomodulatory effects, synthetic methodologies, and critical physicochemical aspects-such as lipophilicity and acidity-significantly underscore the versatility of this *N*-heteroarene. Consequently, the findings presented herein represent an invaluable resource for researchers and clinicians working toward the development of next-generation, quinoline-based antiprotozoal therapies.
